# Open access dataset integrating EEG and fNIRS during Stroop tasks

**DOI:** 10.1038/s41597-023-02524-1

**Published:** 2023-09-12

**Authors:** Zemeng Chen, Chenyang Gao, Ting Li, Xiang Ji, Shuyu Liu, Ming Xiao

**Affiliations:** 1 Institute of Biomedical Engineering, Chinese Academy of Medical Sciences & Peking Union Medical College, Tianjin, 300192 China; 2 Applied Physiology and Kinesiology, College of Health and Human Performance, University of Florida, Gainesville, 32611 USA; 3 Division of Information Science and Engineering, KTH Royal Institute of Technology, MALVINAS VÄG 10, 100 44 Stockholm, Sweden

**Keywords:** Optics and photonics, Psychology

## Abstract

Conflict monitoring and processing are crucial components of the human cognitive system, with significant implications for daily life and the diagnosis of cognitive disorders. The Stroop task, combined with brain function detection technology, has been widely employed as a classical paradigm for investigating conflict processing. However, there remains a lack of public datasets that integrate Electroencephalogram (EEG) and functional Near-infrared Spectroscopy (fNIRS) to simultaneously record brain activity during a Stroop task. We introduce a dual-modality Stroop task dataset incorporating 34-channel EEG (sampling frequency is 1000 Hz) and 20-channel high temporal resolution fNIRS (sampling frequency is 100 Hz) measurements covering the whole frontal cerebral cortex from 21 participants (9 females/12 males, aged 23.0 ± 2.3 years). Event-related potential analysis of EEG recordings and activation analysis of fNIRS recordings were performed to show the significant Stroop effect. We expected that the data provided would be utilized to investigate multimodal data processing algorithms during cognitive processing.

## Background & Summary

Conflict monitoring and processing have always been key characteristics of the human cognitive system. It is closely related to the study of neurological disorders, stroke, and congenital cognitive dysfunction in children^1,2^. Studying how the human brain detects and resolves conflicts is important, and the Stroop task is one of the most widely used methods for this. In 1935, John Ridley Stroop first discovered that when the meaning of a printed word was different from the colour of the word, there would be a cognitive delay, which is the Stroop effect^3^. The Stroop effect makes behavioural responses to incongruent stimuli (when the word’s meaning and the word’s ink colour are not consistent, e.g., the word “blue” shown in red ink) less accurate and slower than responses to neutral stimuli (when the word’s meaning and the word’s ink colour are consistent).

In recent years, there have been several functional neuroimaging methods applied to detect brain activity during the Stroop task, which primarily activates the bilateral frontal lobes^4,5^, such as Electroencephalogram (EEG) and functional Near-infrared Spectroscopy (fNIRS). As for EEG, it records neutral activities with a high temporal resolution, which is within the millisecond range, but has a lack of spatial resolution resulting from volume conduction, thus leading to barriers in source localization^6,7^. The past few decades have seen a rapid increase in the use of functional near-infrared spectroscopy (fNIRS) for monitoring metabolic change in the cerebral cortex, which has excellent spatial but low temporal resolution resulting from the inherent hemodynamic delay^8,9^. Moreover, fNIRS is more robust than EEG when confronted with motion-based muscle activity and electrical noise artifacts^10^. The dual-modal imaging technology that combines the high spatial resolution of NIRS with the high temporal resolution of EEG has gained attention^11–14^. EEG-NIRS correlation analysis helped to further reveal the complex relationship between electrophysiological and hemodynamic changes in neuroscience^15^.

Several public datasets on studying cognitive function recorded fNIRS or EEG signals. One EEG dataset recorded 9 subjects during a verbal working memory task^16^, another EEG dataset contained 50 subjects during visual object processing in the human brain^17^. A public dataset contained 26 subjects who simultaneously recorded EEG and fNIRS data during the N-back task^18^, which is a classical working memory task, and the two signals complement each other in temporal and spatial resolution. However, there remains a lack of public datasets that integrate EEG and fNIRS to simultaneously record brain activity for studying cognitive function. Therefore, we adopted the Stroop paradigm to detect brain activity using fNIRS and EEG simultaneously. It is worth mentioning that our fNIRS data was sampled at 100 Hz.

In the current paper, we recorded 34-channel EEG and 20-channel fNIRS signals of the whole brain simultaneously in 21 healthy participants (9 females/12 males, aged 20–30 years) performing a Chinese colour-word match Stroop task. The high-quality EEG and fNIRS data complement each other in time and space resolution. Compared with the single-modality detection method, synchronous detection provides more effective information in analysing the cognitive function of the human brain.

## Methods

### Participants

A total of 21 healthy volunteers (9 females/12 males, aged 23.0 ± 2.3 years ranging from 20 to 30 years.) were recruited from the Institution of Biomedical Engineering, Peking Union Medical College. Please refer to Table 1 for more details including age, gender, and dominant hand. All participants’ colour vision is normal, and they have no history of mental illness or other related diseases that may interfere with the conclusion of the study. All participants are Chinese native speakers, and the whole experiment paradigm was performed in a Chinese environment. Experimental procedures involving human subjects described in this paper were approved by the Clinical Research Ethics Committee of the First Affiliated Hospital, College of Medicine, Zhejiang University (IIT20210036C-R1). All participants signed informed consent before the experiments and were compensated for their effort by being given a certain amount of test fee.

**Table 1:** Demographic data including age, gender, and dominant hand.

Participant	Age	Gender	Handedness
1	21	female	Right-handed
2	23	female	Right-handed
3	22	male	Right-handed
4	22	male	Right-handed
5	23	male	Right-handed
6	22	female	Right-handed
7	21	male	Right-handed
8	25	male	Right-handed
9	23	female	Right-handed
10	25	male	Right-handed
11	21	male	Right-handed
12	26	female	Right-handed
13	20	male	Right-handed
14	30	female	Right-handed
15	26	female	Right-handed
16	22	male	Right-handed
17	21	male	Right-handed
18	23	male	Right-handed
19	22	male	Right-handed
20	24	female	Right-handed
21	21	female	Right-handed
Average	23.0 ± 2.3	M: 9, F: 12	Right: 21, Left: 0

The average age at the end of this table is presented as mean ± standard deviation.

### Procedures

**Fig. 1:**
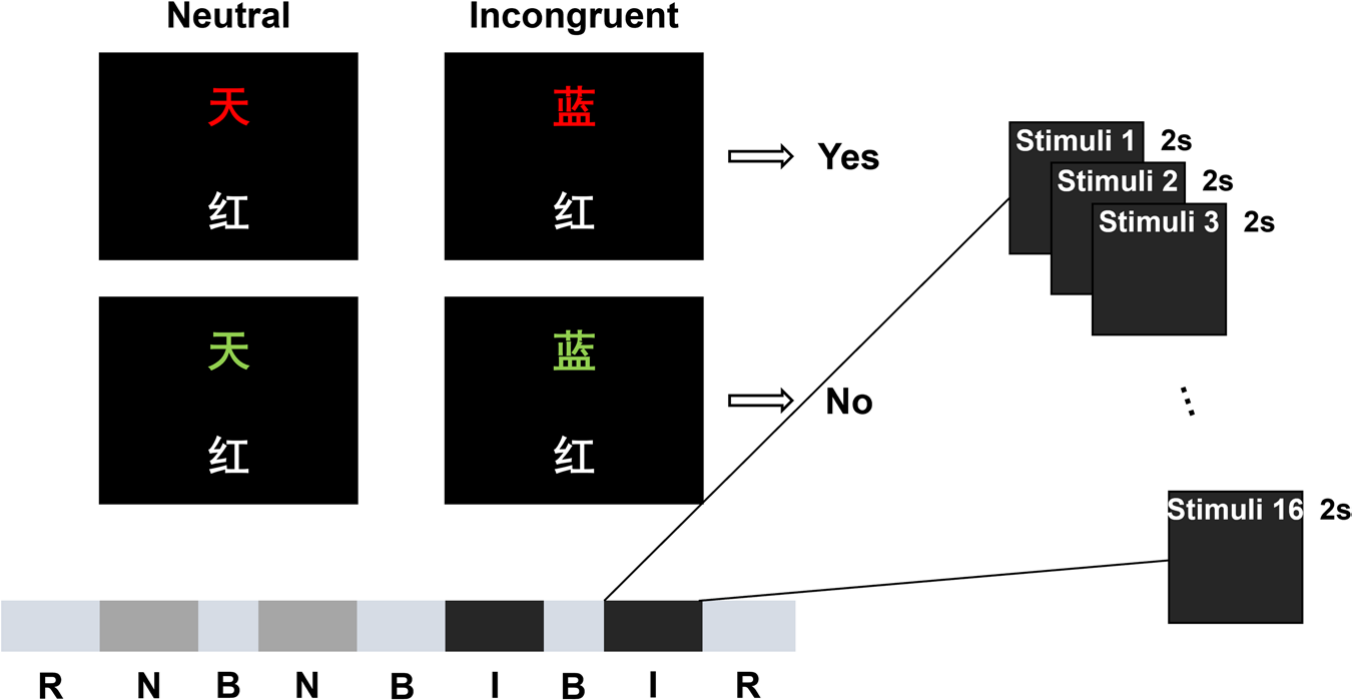
Experimental process. “R” is short for rest, “N” is short for the neutral stimulus block, “B” is short for break, and “I” is short for the incongruent stimulus block.

The classical verbal Stroop colour-word task with a block design was employed in this study, which was adapted from a previous study^4^. Each stimulus contained two Chinese characters and participants was asked to judge whether the colour of the upper one matched the meaning of the lower one. [as Fig. 1 shows]. If the two Chinese characters in the stimulus were corresponding, participants pressed the left mouse button and held it until a trail ended. On the contrary, participants pressed the right button. There were two different kinds of stimulus conditions: neutral and incongruent. In neutral stimuli, the upper Chinese character was a noncolor word which consists of “贯”, “奖”, “放”, “社”, meaning “pass through,” “prize,” “lay,” and “society,” presented in red, yellow, blue, or green; the lower Chinese character was a colour word which consists of “红”, “黄”, “蓝”, “绿”, meaning “red,” “yellow,” “blue,” and “green,” presented in white. In incongruent stimuli, the upper Chinese character was a colour word presented in a different colour. For each stimulus, the numbers of “corresponding” trails and “not corresponding” trails were equal, and those two trails were randomly mixed within each block.

Four blocks were included in this study, they were displayed in order: neutral stimulus block, neutral stimulus block, incongruent stimulus block, and incongruent stimulus block. Each block consisted of 16 trials. During each trial, a stimulus was presented for 2 s, and there are 5 s intervals between two successive trials. There were 30 s rest periods before the first block and after the fourth block. During the break and interval, a white cross was shown in the centre of the screen with a black background. A beep sound appeared half one second before each block to remind participants. There was a practice session before the formal experiment to let participants are familiar with the task. The experimental environment was kept dark and quiet to minimize disturbance to the participants.

### Behavioural data recording

The judgment results of the subjects for each stimulus are collected and recorded by the stimulus program to generate behavioural data.

### EEG recording

The 10–20 system of the international federation was used for EEG recording as scalp sites, with the left mastoid using a Neuroscan 64-channel device (Synamps) to record, while the right mastoid was as a reference. The Electrooculograms (EOGs) were recorded using four additional bipolar electrodes. Two electrodes were placed in the superior and inferior areas of the left orbit to record vertical EOG, and two electrodes were placed lateral to the left and right orbits to record horizontal EOG. A 0.05 to 100 HZ band-pass filter was designed for the EEG and EOG data, then a 50 Hz notch filter is used. The sampling rate of the continuous records is 1000 Hz. A passive Neuroscan cap was used, and the electrode impedances were kept under 5 kΩ.

### fNIRS recording

**Fig. 2:**
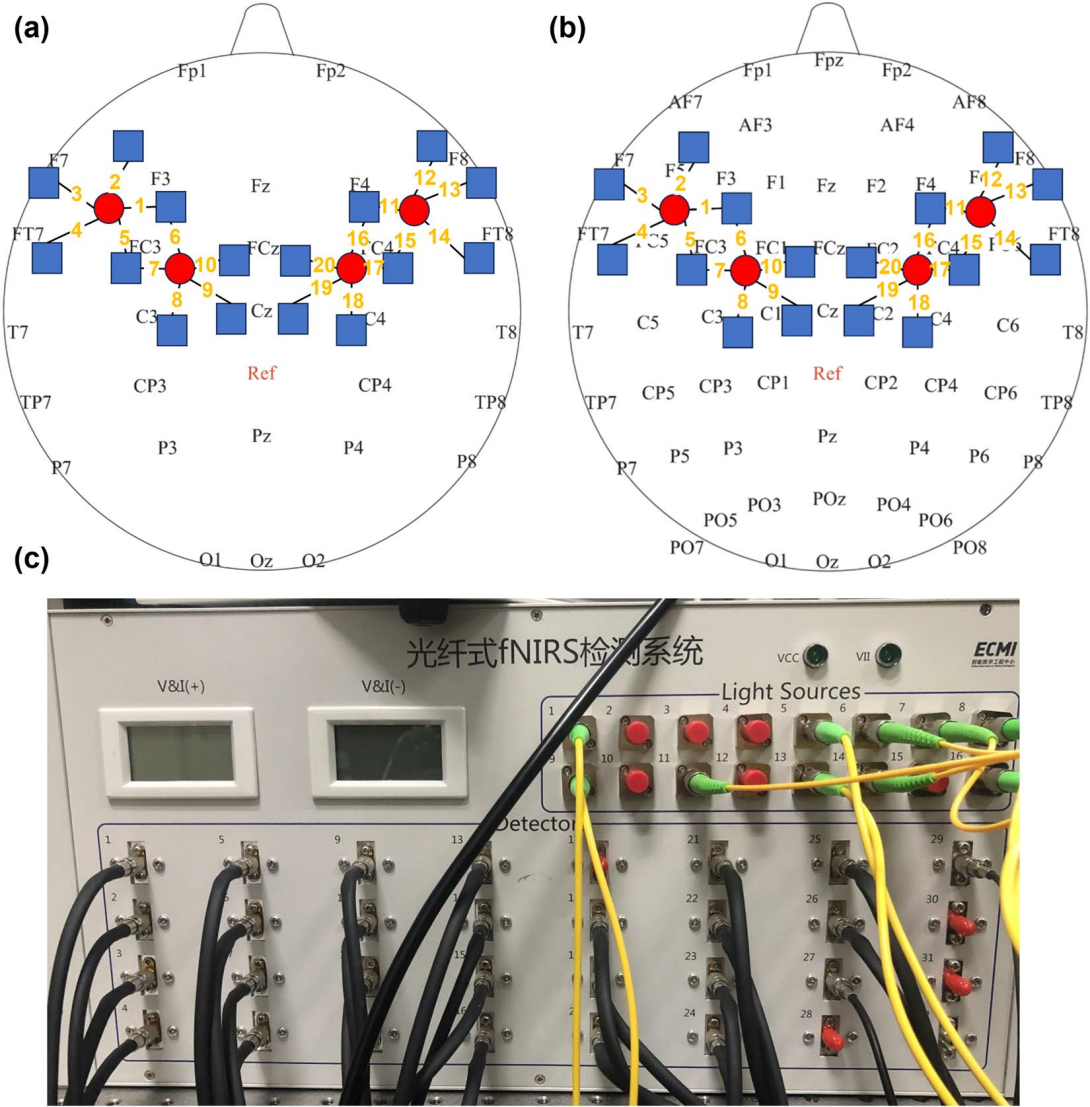
Position arrangement of the fNIRS probe channel of the system and the self-developed fNIRS system. (**a**) fNIRS probe site with the 10–20 system; (**b**) fNIRS probe site with the 10-10 system; (**c**) the self-developed optical fibre fNIRS brain function imaging instrument.

This study used a continuous-wave, modulated light source NIRS system developed by our laboratory [Fig. 2c] to record the fNIRS data^19^. Based on the modified Beer-Lambert Law, two wavelengths (785 nm and 850 nm) were used to determine the concentration changes of HbO (Δ[HbO]) and Hb (Δ[Hb]). The fNIRS probe was placed in the EEG electrode cap and held four sources (represented by the red circle in Fig. 2a,b) and sixteen detectors (represented by the blue square in Fig. 2a,b), providing twenty detector channels covering the frontal and parietal lobes. And the yellow number in Fig. 2a,b was the channel order. There were ten detector channels in both the left and right regions with a 3 cm interval. The sampling rate is 100 Hz, which is far higher than commercial fNIRS systems (around 10 Hz).

During the experiment, the brightness of the lower right corner of the screen was changed with the change of the type of stimuli, which made a photoelectric marking module generate different signals for the fNIRS system and EEG system through a capture card. This made optical and electrical signals can be detected simultaneously, and it was also useful in data processing.

## Data Record

All the raw behavioural data, EEG data, and fNIRS data used in this work are available at Figshare^20^. There are three categories of data in the zip file, including behavioural data, EEG data, and fNIRS data^20^. The behavioural data is saved in the .m file format, the EEG data is saved in the .cnt file format, and the fNIRS data is saved in the .tdms file format. And there is a txt file named “Dataset Description” in the zip file, which explains how to read the three types of data, the meaning of each parameter in the data, how the stimulus type and time of each piece of data correspond, and some details. We present the behavioural data summarized in one file, in .xls file format and .mat file formats, and the EEG data in .set file format after pre-processing. We also present fNIRS raw data exported as .mat file format and fNIRS data after pre-processing in .mat file format. In order to prove the actual sampling frequency is 100 Hz, we provided ‘A set of blank raw fNIRS data to confirm the sampling frequency of this system is 100 Hz’ in .xlsx file format and descriptor of it. A summary of information such as the file name and format are shown in Table 2. For researchers who use python for data processing, we recommend referring to the website (https://www.askpython.com/python/examples/mat-files-in-python) to import data in .mat file format. For researchers using C/C++ programs for data processing, we recommend referring to website (https://www.mathworks.com/help/matlab/matlab_external/reading-a-mat-file-in-cc.html) to import data in .mat file format.

**Table 2:** Summary of datasets.

Folder name	Data format	File size	Description	Participant and paradigm
Raw Behavioral Data In csv	.csv	14.8 KB	Raw behavioral data including stimulus judgment and reaction time	n = 21 9 females/12 males Aged 23.0 ± 2.3 years Chinese native speakers Chinese color-word Stroop Recoding duration (>199 s)
Raw Behavioral Data In mat	.mat	18.1 KB	Raw behavioral data including stimulus judgment and reaction time	
Raw EEG Data In cnt	.cnt	489 MB	Raw EEG data in .cnt file format	
Raw EEG Data In mat	.mat	580 MB	Raw EEG data in .mat file format	
Raw fNIRS Data In tdms	.tdms	191 MB	Raw fNIRS data in .tdms file format	
Raw fNIRS Data In mat	.cnt	192 MB	Raw fNIRS data export as .mat file format	
Pre-processed EEG Data In mat	.mat	163 MB	Pre-processed EEG data (using EEGLAB) in .mat format	
Pre-processed EEG Data In set	.set	163 MB	Pre-processed EEG data (using EEGLAB) in .set format	
Pre-processed fNIRS Data In csv	.csv	126 MB	Pre-processed fNIRS data in .csv file format	
Pre-processed fNIRS Data In mat	.mat	92 MB	Pre-processed fNIRS data in .mat file format	
A set of blank raw fNIRS data to confirm the sampling frequency of this system is 100 Hz	.xlsx	1.33MB	A set of blank raw fNIRS data to confirm the sampling frequency of this system is 100 Hz in .xlsx file format	Blank

## Technical Validation

### Behavioural data processing

All data processing was done using MATLAB R2022b (MathWorks, Natick, MA, USA), statistical tests were analysed using IBM Statistics SPSS 24. For the behavioural data, accuracy, reaction time, and accuracy divided by reaction time (accuracy/reaction time) were calculated for both incongruent stimulus and neutral stimulus. The paired t-test was conducted for paired accuracy, reaction time, and accuracy/reaction time.

### EEG data processing

The EEG data processing toolbox EEGLAB developed by Delorme & Makeig^21^ was employed. Movement artifacts were identified and removed by visual inspection. The EEG data were then re-referenced to channel CZ data. The data were high-pass filtered at 0.5 Hz and low-pass filtered at 45 Hz. Independent component analysis (ICA) matrix was computed, and after that, the movement artifacts and eye-blink artifacts were identified and rejected by visual inspection. The EEG data were then segmented into 1000-ms epochs, including a 200-ms pre-stimulus baseline. After the baseline correction, epochs were averaged separately for incongruent stimulus and neutral stimulus.

**Fig. 3:**
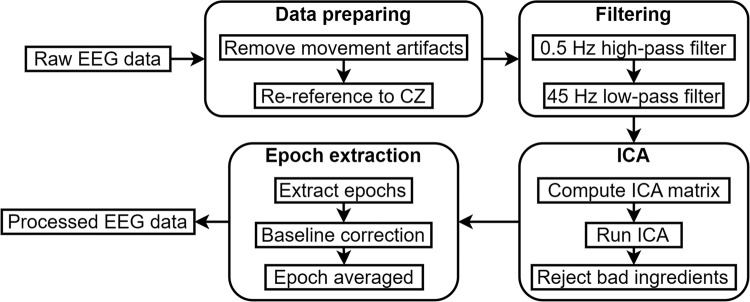
Flow chart of EEG data pre-processing.

Based on our results and previous studies, analyses focused on event-related potentials (ERP) component N200. The N200 ERP is negative component in EEG signals that can be detected during cognitive tasks to executive cognitive control functions^22,23^, and response to word presentation^24^. Each peak N200 amplitude was extracted for both the incongruent and neutral stimuli. The N200 amplitudes and latencies for channel FZ for each stimulus were extracted. Then paired t-tests were used for paired data. Figure 3 is the flow chart of EEG data pre-processing.

### fNIRS data processing

**Fig. 4:**
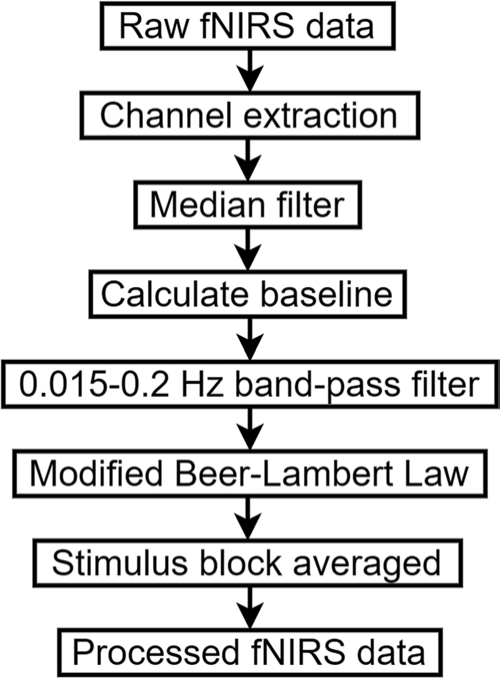
Flow chart of fNIRS data processing.

For fNIRS data processing, HbO and Hb was employed to estimate the changes in cerebral blood oxygenation. According to the marker generated by the photoelectric marking module, raw fNIRS data of the incongruent stimulus and neutral stimulus were divided into two parts. After extracting the incongruent stimulus block and neutral stimulus block, a median filter was used to reduce random noise with additive properties. After the baseline is obtained, a ranged from 0.015 to 0.2 Hz band-pass filter was used to reduce gradual drifts and oscillations of the arterial pulse. Later on, the modified Beer–Lambert law^25^ (MBLL) (1) was used to convert the change in optical density (OD) data into haemoglobin signals.

1 $$O{D}_{\lambda }=\left({\varepsilon }_{Hb{O}_{2}}^{\lambda }\left[Hb{O}_{2}\right]+{\varepsilon }_{Hb}^{\lambda }\left[Hb\right]\right)\cdot DP{F}_{\lambda }\cdot d+G,$$

with a 5.2 of differential pathlength factor (DPF) value at 850 nm and 6.0 of DPF value at 785 nm^26^. At last, the HbO and Hb signals were stimulus block averaged. The mean value of HbO_2_ and Hb signals during the task period (0 to 40 s after the task began) were calculated for the task hemodynamic response for each channel. Then paired t-tests were conducted for paired HbO and Hb data for each channel. Figure 4 is the flow chart of fNIRS data processing.

### Behavioural results

**Fig. 5:**
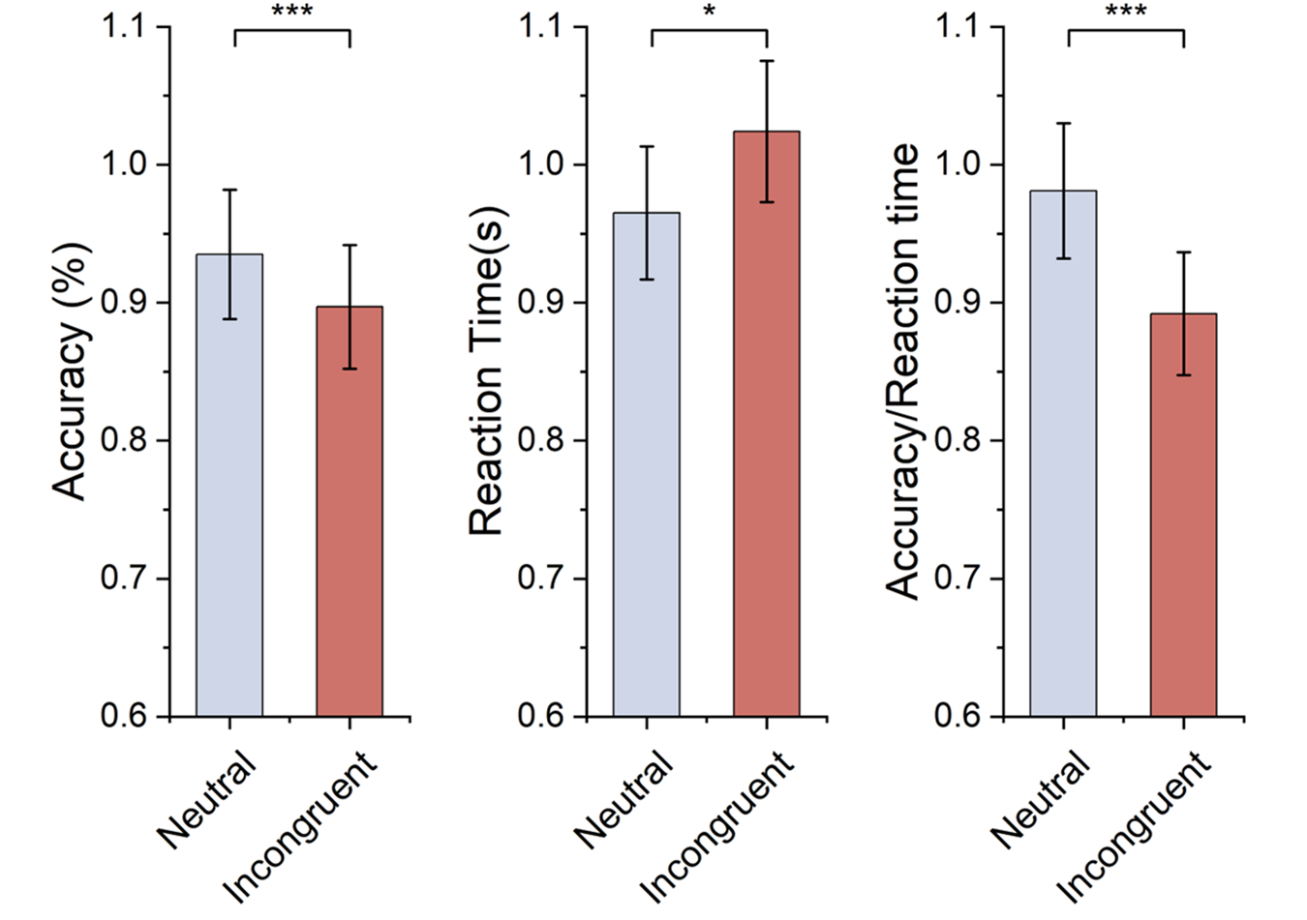
Accuracy, reaction time, and accuracy/reaction time for each stimulus.

Table 3 and Fig. 5 display the average accuracy, reaction time, and accuracy/reaction time for two stimulus conditions. As to accuracy, there was a significant difference between both stimulus conditions, and it was lower for the incongruent condition than the neutral condition. These behavioural results also suggested longer reaction times for incongruent conditions, while the difference was significant. Moreover, the accuracy/reaction time for the incongruent condition is significantly lower. All behavioural results showed significant Stroop effects, which was consistent with previous study^3^.

**Table 3:** The statistical results of accuracy, reaction time, and accuracy/reaction time for each stimulus.

N	Value	Incongruent (M ± SD)	Neutral (M ± SD)	t
21	Accuracy (%)	0.897 ± 0.792	0.935 ± 0.051	2.737***
21	Reaction Time (s)	1.024 ± 0.120	0.965 ± 0.984	−4.282*
21	Accuracy/Reaction Time	0.892 ± 0.157	0.981 ± 0.133	4.227***

T is the t value of paired t-test. The test results in the figure or table as followed abided the standards: *indicates p < 0.05, **indicates p < 0.01, **indicates p < 0.001.

### EEG results

**Fig. 6:**
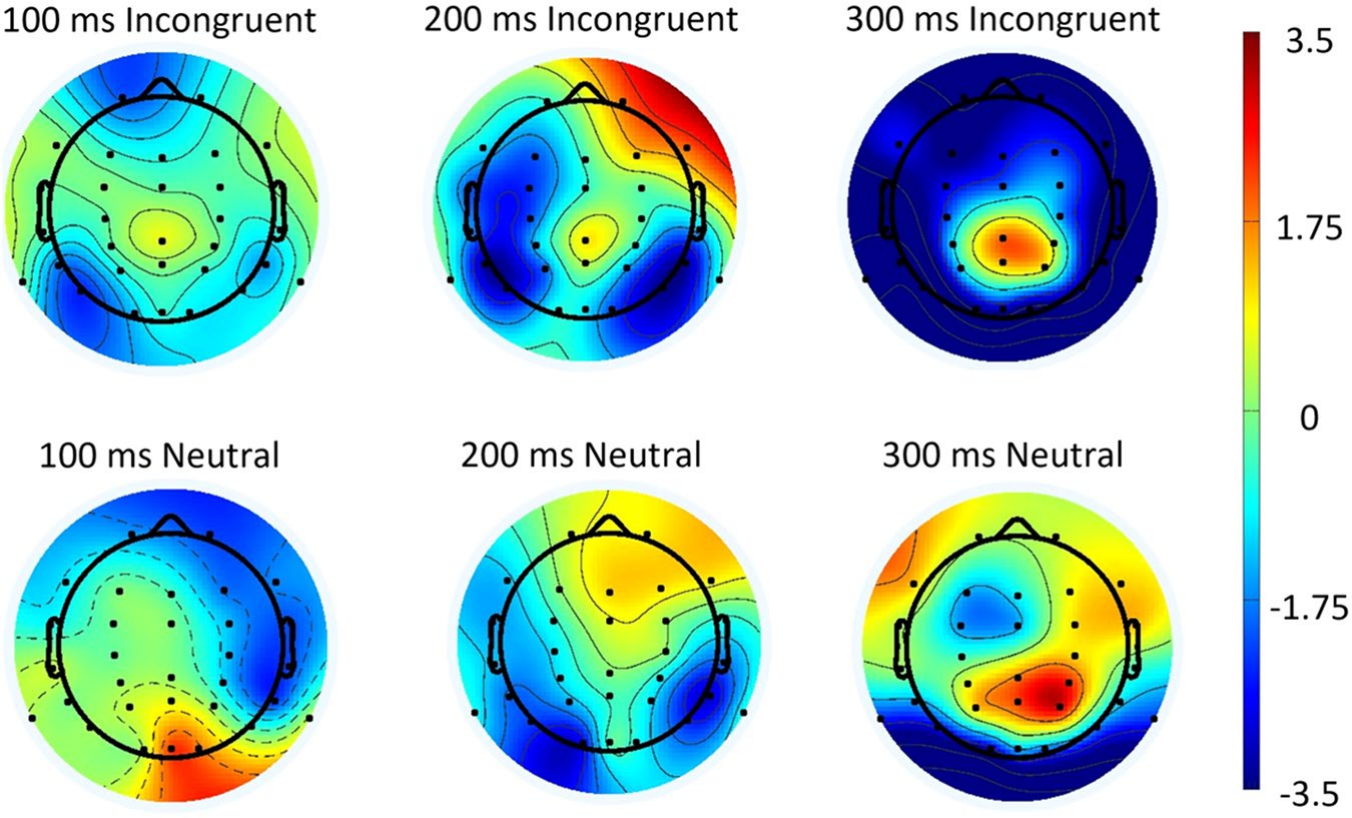
The topographic map at 100 ms, 200 ms, and 300 ms for each stimulus.

As shown in Table 4, latencies and amplitudes of the feature-based components N200 in FZ channel presented significant differences for the incongruent and neutral stimulus. For incongruent stimulus, the latency of N200 in FZ channel was significantly bigger than in neutral stimulus, while the peak of N200 in FZ channel was significantly higher than in neutral stimulus. Figure 6 is the topographic map at 100 ms, 200 ms and 300 ms for each stimulus. In previous studies, the ERP frontal-central N200 component reflects conflict monitoring procedures^21^, which demonstrating that our EEG data recorded brain activity induced by the Stroop effect.

**Table 4:** The statistical results of occurrence time and amplitude of FZ N200 for each stimulus.

Channel	N	Task	Latency (ms)	Amplitude(uV)	t
FZ N200	21	Incongruent	188	−4.764	2.091*
FZ N200	21	Neutral	177	−3.096	2.091*

T is the t value of paired t-test.

### fNIRS results

**Fig. 7:**
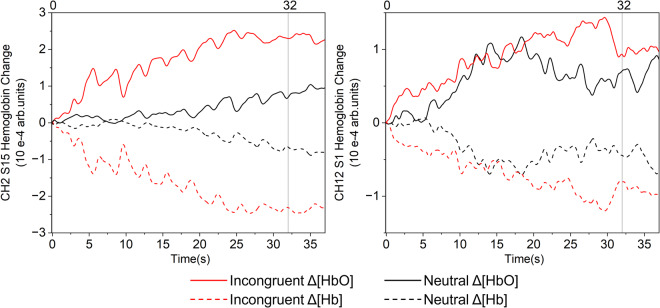
The grand average HbO and Hb signal for each trail at two typical frontal cerebral cortex lobes of a typical participant.

Figure 7 showed the grand average HbO and Hb signals for the incongruent task and the neutral task at two typical frontal lobe channels of a typical participant. The two straight lines perpendicular to the x-axis mark the beginning and the end of the stimuli, and the 32 seconds between the two lines are corresponding to 16 trails of each stimulus. In both CH 2 and CH 1, the activation response for the incongruent task were significantly greater than the neutral task. The t-test p-value of HbO in CH 2 is 0.029, while in CH 12 is 0.024. The p-value of Hb in CH 2 is 0.033, and in CH 12 is 0.017. This significant activation of the prefrontal cerebral cortex was consistent with previous study^12,26^.

## Usage Notes

This dataset simultaneously recorded the 34-channel EEG signals (sampling frequency is 1000 Hz) and 20-channel fNIRS (sampling frequency is 100 Hz) of the whole brain when 21 healthy subjects (9 females/12 males, aged 23.0 ± 2.3 years) during the Chinese colour-word Stroop tasks. The synchronized EEG and fNIRS signals are available for investigating multimodal data processing algorithms during cognitive processing. The data can be used for the construction of an EEG or fNIRS brain network, and can also be used for the analysis of hemispheric lateralization, brain functional connection analysis, effective connection analysis, and the correlation analysis of fNIRS and EEG.

The functional connectivity in fNIRS data undirectedly shows the statistical correlation of activity in different regions of the brain from the viewpoint of functional integration. The Wavelet Transform Coherence method (WTC) can be used to assess intrahemispheric functional connectivity according to previous study^27^. And that study proved that WTC analysis is a reliable method for fNIRS data processing.

The causal relationship between the interactions of different brain regions is reflected by their analysis of effective connectivity on fNIRS data. The effective connectivity analysis describes how information flows transmit between different brain regions. A previous study used Dynamic Causal Modeling (DCM) to analyse effective connectivity, and they used a general linear model (GLM) to identify brain regions that are significantly activated during cognition processing^28^. The Granger Causal mathematical model can be used to analyse effective connectivity between hemispheres^29,30^.

## Data Availability

Scripts to import the fNIRS raw data (.tdms file format) into MATLAB and fNIRS data processing code used above are available at https://github.com/Yaaaaaaaaabby/fNIRS-data-pre-processing-from-Zemeng-Chen.git or https://gitee.com/chen-zemeng/f-nirs-data-pre-processing-from-zemeng-chen.git. A user guide describing the basic situation and usage of the dataset is uploaded together with the code. There are two files in the zip file. The MATLAB code file named “process_fNIRS_EEG_Stroop” is used to pre-process the fNIRS data of a subject. The folder used for MATLAB to load the .tdms file format is named “Matlab_read_tdms_file”. Please add the folder to the MATLAB search path before loading the .tdms file format.
